# Relationships of corticosterone and thyroxine with mortality, mass gain, feeding and activity in Kemp’s ridley sea turtles (*Lepidochelys kempii*) recovering from cold-stunning

**DOI:** 10.1371/journal.pone.0325265

**Published:** 2025-06-18

**Authors:** Kathleen E. Hunt, Elizabeth A. Burgess, Constance Merigo, Adam E. Kennedy, Danielle Dillon, C. Loren Buck, Jodie Treloar, Katherine Graham, Simran Chambers, Teagan Tinuviel, Rosalind M. Rolland, Charles Innis

**Affiliations:** 1 Smithsonian-Mason School of Conservation and George Mason University, Front Royal, Virginia, United States of America; 2 Department of Biology, George Mason University, Manassas, Virginia, United States of America; 3 Wildlife and Ocean Health Program, Anderson Cabot Center for Ocean Life, New England Aquarium, Boston, Massachusetts, United States of America; 4 Rescue and Rehabilitation Department, New England Aquarium, Boston, Massachusetts, United States of America; 5 Department of Biological Sciences, Northern Arizona University, Flagstaff, Arizona, United States of America; 6 Animal Health Department, New England Aquarium, Boston, Massachusetts, United States of America; Tshwane University of Technology, SOUTH AFRICA

## Abstract

Mass strandings of juvenile Kemp’s ridley sea turtles (*Lepidochelys kempii*) occur annually on the shores of Cape Cod, Massachusetts, USA, during the months of Oct-Dec. Strandings have increased from dozens to hundreds per year in the past two decades, challenging recovery and management of this critically endangered species. Most stranded turtles are suffering from “cold-stunning”, a life-threatening hypothermia-like condition, and are brought to nearby marine animal veterinary clinics for treatment and rehabilitation. Though most individuals survive, some mortality does occur, and even among surviving turtles there can be prolonged deficits in health and behavior. Previous studies have indicated that upon admission, the adrenal stress hormone corticosterone is elevated approximately an order of magnitude above presumed baseline, while plasma thyroxine is often undetectable, suggesting that these two hormones show promise as markers of recovery from cold-stunning. In this prospective study, 106 cold-stunned Kemp’s ridleys were monitored during rehabilitation, with serial blood sampling at 0, 3, 7, 18, 30, 60 and 80 days post-admission to compare plasma concentrations of corticosterone and thyroxine to mortality, mass gain, feeding and activity. Corticosterone and thyroxine normalized in 88% of turtles by approximately day 18, but 12% showed persistent elevations of corticosterone (typically 2-3x above baseline), and persistently low thyroxine. Elevated corticosterone at day 18 was found to be predictive of mortality after day 18. The endocrine profile of high corticosterone and low thyroxine is also associated with lower rates of gain in body mass over time and reduced feeding. As prolonged deficits in growth affect body size at release, low mass gain may affect the predation risk on these juvenile turtles subsequent to release. These results suggest that endocrine biomarkers are useful for monitoring recovery of turtles in rehabilitation, and that growth rates and mass gains during rehabilitation may warrant further investigation.

## Introduction

Mass strandings of juvenile Kemp’s ridley sea turtles (*Lepidochelys kempii*) occur annually on the shores of Cape Cod, Massachusetts, USA, during the months of Oct-Dec, coinciding with the autumnal drop in sea surface temperature in Cape Cod Bay [1,2]. In the past two decades, annual strandings have increased from dozens to hundreds, thought to be related to altered seasonal migration and foraging patterns of this species due to warming sea surface temperatures in the North Atlantic [2]. As the Kemp’s ridley is a critically endangered species [3], these mass strandings present a challenge for conservation of the species [2], especially since this species has still not recovered its original population growth rate since the 2010 Deepwater Horizon oil spill [4–6]. Therefore, for conservation purposes as well as individual animal welfare, there has been a concerted research effort to determine how best to treat, rehabilitate, and release stranded turtles [7–12].

Kemp’s ridleys that strand in Cape Cod Bay are usually suffering from “cold-stunning,” a hypothermia-like clinical condition that involves prolonged reductions in body temperature. Affected turtles are prone to secondary clinical abnormalities including dehydration, electrolyte derangements, acidosis, pneumonia, sepsis and traumatic injuries [7–9,13]. Most cold-stunned Kemp’s ridleys found on Cape Cod are initially admitted to the sea turtle veterinary hospital of the New England Aquarium (NEAq), located in Quincy, MA, USA, where they undergo comprehensive evaluation, individualized veterinary treatment and long-term rehabilitation. Once turtles are stabilized, they may then be transferred to other wildlife veterinary clinics for the remainder of their rehabilitation, but several dozen typically remain at the NEAq clinic for a period of four to eight months for long-term rehabilitation. The majority (>70%) of cold-stunned turtles admitted to the NEAq sea turtle clinic are rehabilitated and are released the following spring or summer, but on average ~20–30% of these rare turtles die (e.g., [14]). Several biomarkers have been identified that can help predict poor prognosis in the first few weeks of clinical care when mortality is highest [8,9,13,14]. However, some mortality occurs weeks to months into rehabilitation due to chronic pathologic conditions, by which time most other turtles are swimming and eating well (e.g., [15]). Further, even among surviving turtles, there are individual cases of illness and prolonged abnormalities in behavior or physiology that may last months. Gaps in knowledge remain about patterns in, and predictors of, “late mortality” (i.e., mortality >2 weeks after admission) as well as causes or predictors of other aspects of slow recovery in surviving turtles. Preliminary studies [10] indicate that two hormones, corticosterone and thyroxine, might help identify turtles at risk of late mortality or slow recovery, and could be useful indices for understanding, predicting, and potentially ameliorating risk of late mortality or prolonged deficits in health.

Corticosterone (“Cort”) is the major adrenal glucocorticoid “stress hormone” of turtles and most other non-mammalian vertebrates. Sea turtles, like most vertebrates, respond to a variety of stressors (e.g., cold-stunning, entanglement, capture, injury, transport, oiling; [10,11,16–22]) with increases in circulating Cort, which coordinates a systemic “stress response” that increases fuel availability (e.g., gluconeogenesis, lipolysis), alters behavior (e.g., increased foraging, increased spatial movement), has complex impacts on immunity (short-term stimulation followed by long-term suppression), and temporarily suppresses nonessential processes such as growth and digestion [23,24]. In the short term, this physiological stress response is considered adaptive, in that it redirects available energy toward coping with the stressor [24]. However, prolonged or repeated elevations in Cort can have numerous negative long-term effects, including reduced wound-healing time, vulnerability to infections, and inhibition of growth and reproduction [24]. In newly admitted cold-stunned Kemp’s ridleys, Cort is elevated by approximately an order of magnitude above presumed baseline (<5 ng/mL; [10,18]; also see [20]), often peaking at around 30–50 ng/mL, with some cases over 70 ng/mL, among the highest plasma concentrations reported for this species [10]. This extremely elevated initial Cort then typically drops below 10 ng/mL after two weeks in recovery, by which time turtles are assumed to have largely adjusted to the clinical environment [10,18]. However, Cort may remain mildly elevated (e.g., 6–10 ng/mL). A question of interest, therefore, is whether prolonged mild elevations in Cort might affect behavior and physiology even in those turtles that survive. In vertebrates generally, prolonged stress in juvenile life stages can reduce growth rate, often with long-lasting effects on later adult body size and reproduction [25,26]. Low growth rate and consequent smaller body size may be a particular concern for aquatic species such as sea turtles, as many aquatic predators are gape-limited (limited by the size of their mouth) and preferentially target small prey [27–29]. Cort also often influences feeding behavior and activity, which together affect caloric intake and expenditure, influencing energetic balance and growth [24,30]. Thus, investigation of the potential relationships of Cort with growth, feeding and activity is warranted. Finally, in addition to Cort’s potential direct effects on growth and other aspects of behavior and physiology, it may be useful as a biomarker broadly reflective of physiological status.

The thyroid hormone thyroxine (“T4”) may also be a biomarker of interest in cold-stunned Kemp’s ridleys, as it shows an opposite pattern to Cort: plasma T4 concentrations of cold-stunned Kemp’s ridleys tend to be undetectably low on admission, and rise to presumed-normal concentrations (~1 pg/ml) after two weeks in recovery [10]. T4 is an inactive form of thyroid hormone released by the vertebrate thyroid gland, subsequently converted by target tissues to the active hormone tri-iodothyronine (“T3”). Together termed the “thyroid hormones”, T4 and T3 have been best studied in endotherms, in which they directly determine basal metabolic rate and tend to stimulate feeding, activity and growth [31,32]. Though less studied in ectotherms, the ectothermic vertebrates do also have thyroid glands (or scattered thyroid glandular tissue) and produce both T4 and T3, which seem to have generally similar functions as in endotherms, stimulating feeding, growth, and activity (i.e., opposite effects compared to Cort) and influencing metabolic rate (reviewed in [32,33]). In both endotherms and ectotherms, prolonged stress can cause declines in circulating T4; in fact, endocrine activity of the thyroid gland can be directly inhibited by Cort, particularly if the stressor involves reduced body temperature, starvation, or both [34–36]. T3 is typically below detectability in turtle plasma, but T4 is readily detectable (e.g., [10]), including the biologically relevant fraction of T4 known as “free” T4 (fT4; T4 that is unbound to plasma carrier proteins). Available data indicate that fT4 concentrations are suppressed by stress and/or cold water exposure in sea turtles. For example, experimental exposure of juvenile Kemp’s ridley sea turtles to cold water causes dramatic declines in plasma fT4 concentration, accompanied by reductions in feeding and activity [37]. Like Cort, fT4 often influences feeding and activity, with data from other ectotherms suggesting a bidirectional relationship in which low feeding and low activity both contribute to, and are exacerbated by, low fT4 [38–41]. In sum, these patterns suggest that Cort and fT4, together, may be useful biomarkers for monitoring turtle recovery from cold-stunning and assessing patterns in prolonged deficits in growth, feeding and activity.

To further examine the potential utility of Cort and fT4 for predicting late mortality or prolonged deficits in growth, feeding and activity, we undertook a prospective correlational study of 106 cold-stunned juvenile Kemp’s ridley turtles over multiple months of rehabilitation, during which we monitored clinical status, Cort, fT4, feeding, clinical notes on activity, and body mass (to calculate mass gain, a proxy of growth) for at least 60 days after initial admission. Specific study goals included: (1) determining typical patterns of Cort and fT4 beyond the first two weeks of recovery, including re-evaluating “presumed normal” ranges with a larger dataset than was previously available; (2) investigating whether either hormone, at the two-week time point, can be used as a predictor of late mortality, i.e., mortality that occurs after two weeks post-stranding; (3) evaluating relationships with Cort and/or fT4 with concurrent (same week) feeding behavior and activity, and also with subsequent growth rate assessed later across multiple months of rehabilitation; and finally, (4) investigating whether endocrine patterns differed across the three study years, given that one of the study years proved to have an exceptionally high (record-breaking) number of turtle strandings. Our overall goal was to identify and evaluate biomarkers that could be useful for monitoring rehabilitation or identifying individuals at risk, in order to improve the long-term health and survival of rehabilitated sea turtles.

## Materials and methods

### Ethics statement

Ethics approval was granted by the Animal Care and Use Committee of the New England Aquarium (IACUC protocol 2012-04). The NEAq sea turtle rehabilitation program and associated research were conducted with the authorization of the United States Department of the Interior Fish and Wildlife Service (permit number TE-697823), and followed the United States Fish and Wildlife Service Standard Permit Conditions for the Care and Maintenance of Captive Sea Turtles ([42,43]; see also [44]). Our research complied with all other applicable federal, state, local, and institutional guidelines for ethical use of animals in research. (Note that informed consent guidelines were not applicable, as our research was restricted to wild animals that were not privately owned.) Veterinary and husbandry personnel were trained following NEAq’s internal training program and were approved and authorized by the United States Fish & Wildlife Service as per the New England Aquarium’s sea turtle rehabilitation permit requirements. Additionally, all research personnel involved in the clinical phase of the study (2012–2015) completed animal care training through the CITI program of University of Miami.

### Study subjects

All 106 study subjects were juvenile Kemp’s ridley turtles that were admitted to the New England Aquarium’s sea turtle rehabilitation center (“NEAq”; Quincy, MA) during 2012–2014 (Table 1). All turtles were found stranded in a cold-stunned state on the northern shore of Cape Cod, MA, during fall months of the three study years (admission dates during October-December of 2012–2014) and were rehabilitated for multiple months, with survivors typically released back to sea during the following spring (e.g., April-June of 2013–2015). All turtles were estimated to be 2–3 years of age based on carapace length at the time of stranding compared to known-age conspecifics (B. Higgins, National Marine Fisheries Service, pers. comm.; [45]). Clinical data for any given date are expressed as days since that turtle was found stranded (“days since stranding”), with day 0 being the day the turtle was found stranded on Cape Cod. Turtles found early in the day were typically admitted to NEAq in the afternoon of day 0, while turtles found later in the day were typically triaged locally on Cape Cod on day 0 and transported to NEAq the next day, i.e., admitted to the NEAq clinic on day 1. In any given study year, ~ 30–40 admitted turtles were assigned to the study by simple random allocation, with the goal of attaining a total sample size of ~100 turtles with near even representation across three different stranding years (e.g., stranding seasons of fall 2012, fall 2013, and fall 2014). Total study duration was 972 days across all three study years.

**Table 1:** Sample sizes of cold-stunned juvenile Kemp’s ridley sea turtles (*Lepidochelys kempii*) assessed in this three-year prospective study.

	Year 1 2012−13	Year 2 2013−14	Year 3 2014−15	ALL YEARS COMBINED
Turtles that survived	29	19	32	**80**
Turtles that died	12	9	5	**26**
Total # turtles studied	41	28	37	**106**
Samples from survivors	123	101	246	**470**
Samples from turtles that died	41	14	23	**78**
Total # samples assayed	164	115	269	**548**
*Mortality details*				
Mortality timing:				
Early mortality (before day 14)	9	7	0	**16**
Late mortality (after day 14)	3	2	5	**10**
Natural death vs. euthanasia:				
Natural death	10	8	4	**22**
Euthanasia	2	1	1	**4**

Our clinical treatment of cold-stunned Kemp’s ridley turtles has been fully described elsewhere [7,12]. Individual turtles were assigned a unique identification number which was also recorded on the carapace (paint pen) as well as a numbered forelimb “bracelet”. Details of medical interventions varied for each patient based on veterinary evaluation and were tailored to meet individual patient needs, often including fluid therapy, cardiorespiratory support, antimicrobial therapy, analgesia and anti-inflammatory drugs, and nutritional support. Briefly, turtles were gradually rewarmed during their first week of hospitalization while medical problems were treated. Once rewarmed and swimming well (typically by day 7), turtles were placed in large tanks with other similar-sized turtles, with individualized veterinary treatment continued as necessary. Turtles were subsequently maintained in saltwater tanks at ~26°C (the average temperature range for this species; [46]) as described in [10], with twice-daily health monitoring by clinical staff and twice-daily feeding (herring and squid). Food was offered by tongs to individual turtles by trained personnel so that food intake could be recorded. Under unfamiliar conditions of human care, turtles often initially required patient, gentle, persistent food offering, but, once healthy, turtles began to recognize human-offered foods quickly and thereafter fed readily. Most turtles contributed data to our study up to day 80 of rehabilitation, after which many turtles in stable condition were transferred to other institutions for continued care until release the following spring. In Year 3 of the study, a subset of study turtles were selected at random to be retained at our institution for further study past day 80 in order to monitor longer-term patterns in feeding and body mass. While in most cases no follow-up is available after surviving turtles are released back to sea, all released turtles are routinely equipped with Inconel flipper tags and a passive integrated transponder (PIT) to enable subsequent identification if detected by a human observer,

### Study endpoints and euthanasia

Due to the endangered status of this species and federal permit regulations, stranded cold-stunned patients are medically managed with hope of rehabilitation. The majority survive, but approximately 20–30% die due to physiologic and pathologic sequelae while undergoing medical management [8,12,14]. During the course of this study, 106 patients were enrolled, of which 22 died naturally and four were euthanized. Causes of death included severe physiologic derangements secondary to cold-stunning and stranding (*n *= 13; [9,13,14]); chronic severe infection (*n* = 11; [8,15]); and multi-organ inflammation and dysfunction (*n *= 2). Euthanasia, when indicated, was achieved by deep sedation followed by intravenous injection of pentobarbital immediately upon reaching a euthanasia decision. Euthanasia decisions were made by the attending veterinarians following the United States Fish and Wildlife Service Standard Permit Conditions for the Care and Maintenance of Captive Sea Turtles [42,43], “if an illness or injury is terminal or untreatable” (see also [44]). Death was confirmed by attending veterinarians based on absence of cardiac function as assessed by echocardiography, as well as physical examination findings such as rigor mortis and absence of neuromuscular reflexes. When necessary, intravenous or intracardiac potassium chloride was used to terminate residual cardiac function.

### Early vs. late mortality

Mortalities were classed either as “early mortality”, defined as mortality before day 14 (the time period when mortality is most common), or “late mortality”, defined as mortality occurring on or after day 14. Most turtles had one blood sample taken on or near day 18 for routine clinical assessment (this timing is determined by medication schedules and clinical logistics), which in all but one case was before any late mortality occurred. Cases of late mortality were compared to surviving turtles to evaluate whether corticosterone or thyroxine from the day 18 blood sample are predictive of late mortality (see Data Analysis).

### Clinical examinations, body mass and blood sampling

Turtles periodically experienced clinical examinations and blood collection for routine clinical care, from which this study obtained data on body mass as well as plasma samples for hormone measurements. During clinical examinations, turtles were gently removed from the water with a hand-held net, slightly tipped for ~5 seconds to allow water to drain from the mouth, and then placed in a transport container and carried to an examination room. If a blood sample was to be collected that day, 2–3 mL of whole blood was immediately collected from the external jugular vein into a heparinized tube as previously described [9], followed by other clinical assessments (not presented here) and lastly weighing of the turtle on a digital scale to the nearest 0.1 kg, after which the turtle was gently returned to its tank. Because blood sampling and associated handling can themselves increase Cort in wild vertebrates, care was taken to minimize the time taken to collect the blood sample (“bleed time”), defined as the elapsed time between first disturbance of the turtle when the net entered the turtle’s tank, and collection of the blood sample. Due to clinical constraints and caseload, it was not always feasible to precisely measure bleed time, but in those cases where timing was possible, bleed time was generally <3 min, was always <10 min, and total handling time was typically <10 min as well. In this species, bleed times of <10 min have no detectable effect on Cort data [18]. Immediately after blood sample collection, whole blood was centrifuged in an on-site laboratory with plasma pipetted to a cryovial for storage at −80°C until assay.

During the first two months of rehabilitation, turtles were examined and weighed every three days. Examinations then declined in frequency to weekly or monthly depending on individual medical needs. Blood sampling occurred only during certain clinical examinations, typically on admission (day 0 or day 1), day 3, day 18, and approximately monthly thereafter, until transfer to another institution (often at ~80 days), although sampling was more frequent in some cases when medically necessary. For this study, minor deviations of 1–2 days in sampling schedule (e.g., blood collected on day 58 instead of day 60) were ignored. The sampling schedule after day 18 varied each year based on clinical needs and caseload, but all years included a blood sample on day 60, and typically on day 80 as well. As a result, many of our analysis metrics (e.g., mass gain) track turtle data up to day 60 and/or day 80. In Year 3, the subset of turtles that were studied for an extended period also had day 108 (~3.5 months) blood samples and, if not yet released back to sea, day 140 (~4.5 months) blood samples. Thus, our study includes blood sampling time points and associated clinical data for days 0–1, 3, 18, 60, 80, 108, and 140 (though with much lower sample sizes for the last two time points).

### Hormone assays

Plasma samples were assayed for Cort and fT4 using double-antibody ^125^I radioimmunoassay kits that had previously been validated for Kemp’s ridley turtle plasma ( [10]; Cort, catalog #07–120103; fT4, catalog #06B-257214; MP Biomedicals, Solon, Ohio, USA). No extraction solvent was employed, i.e., plasma samples were assayed unextracted (aka “direct”), due to the aforementioned validations indicating good assay accuracy with plasma of this species. An assay specific for fT4 was selected as the most informative measure of thyroid function (i.e., rather than alternative assays for T3, total T4, or thyroid-stimulating hormone) due to prior studies indicating that the fT4 assay has superior detectability, antibody binding, and biological relevance for this species [10]. For both the Cort and fT4 assays, an additional low standard (created via two-fold dilution of the manufacturer’s lowest standard in assay buffer) was added to the standard curve to improve measurement precision at low concentrations. Based on previous experience with typical hormone concentrations in this species, the Cort assay was run at half-volume with turtle plasma samples diluted 1:10 in assay buffer, while the fT4 assay was run at full volume with all samples at 1:1. Non-specific binding tubes and blanks were assayed in quadruplicate, and standards, controls, and samples in duplicate. Any samples with a coefficient of variation >10% between duplicate tubes, or that fell outside 10–90% bound, were rediluted and reassayed accordingly. Intra- and inter-assay variations for both assays were <10% in this study. For further assay details, including antibody cross-reactivities, sensitivities and precision testing, see Hunt et al. [10,11,18].

### Mass gain

The metric of growth for each turtle was based on gain in body mass occurring during rehabilitation, calculated as percentage gain from day 7 to day 60. Day 7 was used as the initial starting mass rather than day 0 or day 1 due to the fact that turtles are usually dehydrated on admission and also anorexic during their first ~5–7 days in the clinic, and thus any mass gain during the first week would primarily be due to therapeutic and voluntary rehydration. Additionally, turtles are typically not offered food until day 7. Some other metrics of growth were sporadically available in clinical charts, e.g., carapace length and carapace width, but these measurements were not consistently available for all turtles, and thus our analysis focuses on percentage gain in body mass.

### Feeding

To assess feeding behavior as a potential prognostic indicator of later growth, and to examine the relationship of feeding with near-simultaneous hormone concentrations, turtle feeding behavior was examined across days 11–17 and was then associated with the day 18 hormone data. Feeding was assessed across a seven-day timeframe due to the fact that turtle feeding can be erratic day-to-day (e.g., a turtle may eat a large amount on one day and relatively little the next day), whereas weekly mean food intake is relatively consistent (KEH, pers. obs.). Feeding was evaluated for the week prior to blood collection, rather than the week during or the week after, due to potential effects of handling stress on subsequent feeding behavior.

To assess feeding, every turtle was individually offered pre-weighed pieces of herring and/or squid to satiety during twice-daily feedings, with the total mass of food eaten recorded for each turtle for each day. Feeding behavior was then quantified for each individual turtle in two ways: mean mass (g) of food eaten per day (averaged across one week), and feeding consistency, defined as whether or not the turtle consistently ate voluntarily every day that week (Y/N). For the first feeding metric (mean g of food eaten per day), the total mass of ingested food was recorded for each turtle for each day, and at the end of the week, mass of food eaten per day was averaged over the seven days. For the second feeding metric of feeding consistency, turtles were categorized as either having voluntarily eaten at least one food item on all seven days (Y) or having had at least one day with no food eaten (N). A few days on which feeding data were not collected due to staffing limitations were excluded from the dataset.

### Activity

During daily checks of every turtle, experienced clinicians recorded notes on each turtle’s general activity and behavior, particularly noting any turtles that were not actively swimming. While these clinic notes represent only brief snapshots of each turtle’s daily activity, we utilized them for exploratory assessment of potential endocrine-behavior relationships in this species, as follows: Each turtle’s activity notes in clinical charts for the seven days prior to the day 18 blood sample were summarized with a single categorical dichotomous variable, with the turtle classed either as active every day (Y) or not active every day (N). Clinical records of actively swimming turtles typically include the terms “BAR” (bright, alert and responsive) or “A4F” (using all four flippers). “Inactive” turtles were thus defined as those whose case notes for that day did not include “BAR” or “A4F”, and instead included any of the following three terms: “QAR” (quiet, alert and responsive), “ROB” (resting on bottom of tank), and/or “Quiet”. If a turtle was classed as Inactive on any day that week, the turtle was classed as “not active every day” (N); otherwise (no “Inactive” days), the turtle was classed as “active every day” (Y). Hormone concentrations from the day 18 blood sample were then associated with the activity status of the turtle for the week prior to blood sampling.

### Data analysis

#### Descriptive statistics and data management.

Data were summarized using mean ± S.D. Cort and fT4 concentrations were transformed using the common logarithm, log_10_, to correct for a skewed, non-normal distribution. Levene’s tests were conducted for all variables to check for homogeneity of variance, with Welch’s correction applied for unequal variances. Many samples had undetectable fT4 concentrations (n = 133 out of 462 samples measured), particularly those initial samples taken upon admission (n = 60 out of 103), that fell below the limit of assay detection of 0.46 pg/mL. For statistical analyses of fT4 data, these datapoints were assigned a nominal value that was half of the assay quantification limit (i.e., 0.23 pg/mL, representing the midpoint of the range of possible hormone concentrations in such samples) to avoid non-random missing data in statistical analyses [47]. Mortality rates of turtles in different years were investigated using χ^2^ analysis to compare the frequencies of individuals that survived and those that died across each of the three study years. All clinical data were inspected by a veterinarian (CI) for any clinically relevant deviations from expected values for healthy individuals (as compared to data reported for juveniles of this species in [7,13,18,48–52]).

#### Initial hormone concentrations.

Initial hormone concentrations of turtles upon admission were examined using a general linear model (GLM), fitted by maximum likelihood with a normal distribution and an identity link function. A main effect model was used to evaluate the difference between study year and final survival outcome for the turtle (survived until release, vs. did not survive to be released) in relation to initial Cort and fT4 values upon admission to hospital.

#### Patterns of hormones across rehabilitation.

To examine hormone concentration patterns throughout rehabilitation (up to 140 d), we used a linear mixed model (LMM) to assess the influence of sampling time points (day 0–1, 3, 18, 60, 80, 108, 140) on Cort and fT4 concentrations (response variables) in all turtles. Individual turtle identification and the year of stranding (2012, 2013, 2014) were included as covariates in the model to account for repeated measures and individual-level variability. The year of stranding was treated as a random effect, as it represents a grouping factor that may account for unmeasured environmental or procedural variation across years, rather than acting as a direct explanatory variable influencing hormone concentrations.

#### Hormones and mortality.

We performed binary logistic regression to predict the probability of turtle survival based on predictor variables (Cort and fT4) at clinical time points early in rehabilitation, specifically focusing on day 0–1, day 3, and day 18 hormone data as potential predictors of subsequent clinical outcomes. A stepwise approach was employed to evaluate hormone concentrations (explanatory variables) at each clinical time point and identify when they might predict survival outcomes (dependent variable) for turtles. Model performance was assessed using several key metrics, including Omnibus test to evaluate whether the model significantly improves upon the null model, Akaike Information Criterion (AIC) for model comparison, −2 Log likelihood (−2LL) to assess model fit, and Nagelkerke *R*^2^ to measure the proportion of variance explained by the model. For time points where hormone data proved to be significant predictors of clinical outcomes, discriminant function analysis was performed to determine the probability of group membership (i.e., survived or did not survive to be released) based on hormone concentration at that time point, as identified through binary logistic regression. To estimate hormone concentrations associated with the highest risk of turtle mortality, a logistic function was fitted to the probability of group membership as a surviving turtle based on hormone concentration at that time point.

#### Body mass.

Analysis of variance was used to evaluate whether the initial body mass of hospitalized turtles (measured on day 7) varied across the three years of the study (2012, 2013, 2014). Pearson correlation analyses were performed to assess the strength and direction of the relationship between initial body mass and percentage gain in mass after 60 d, as well as between hormone concentrations at each clinical time point and the percentage increase in body mass of surviving turtles from day 7 to day 60. Next, we used GLM to examine the influence of potential predictor variables from day 18 on turtle mass gain during rehabilitation (i.e., total percentage increase in body mass across days 7–60). This analysis included day 18 hormone concentrations, feeding consistency (Y/N), amount of food ingested (g/d), and activity (Y/N). Based on the outcomes, curve estimation models were used to further explore the relationship between mass gain and hormone values on day 18. *R*^2^ values were used to evaluate the fit of the models, with the highest *R*^2^ value indicating the best fitting model. Although a full analysis of individual turtle mass gain trajectories is beyond the scope of this study, the mass gain curves for each individual turtle were visually examined to inspect the slope and identify any plateaus in growth.

#### Relationship of hormones with feeding and activity.

A GLM was used to evaluate the relationship between observed consistent feeding and active swimming behaviors (both binary variables: Y/N), the amount of food ingested (g), and Cort and fT4 concentrations in turtles on day 18.

All analyses were conducted using IBM SPSS Statistics (version 28.0.0 for MacOS) and GraphPad Prism (version 10.0.3 for MacOS). Assumptions were tested by visually inspecting residual distributions and Q-Q plots. A probability value of p < 0.05 was considered significant for all statistical tests. After conducting initial GLM analyses to assess significant differences in initial hormone concentrations, feeding and activity, and body mass gain, we applied Bonferroni p-value corrections for post-hoc multiple comparisons to identify which specific group pairs differed significantly, adjusting for the increased risk of Type I errors.

## Results

### Mortality

Of the 106 turtles in this study, 80 (75.5%) turtles survived to be released to the wild, and 26 (24.5%) died (Table 1). Mortality rate was similar across all three years for the turtles in our study (χ^2^ = 3.80, *p* = 0.15) (Table 1). Of the 26 mortalities, 16 (62% of mortalities) occurred within the first 14 days after stranding and were considered early mortalities (Table 1), most of which occurred in the first week (14 of 16 cases occurring during days 0–7). The remaining ten cases (38.5% of mortalities) died after 14 days and were considered late mortalities (Table 1).

While in most cases no follow-up was available on surviving turtles after they were released back to sea, two of the released turtles were encountered alive and healthy in subsequent years: (1) Turtle 12–056, originally stranding in late 2012 and released on April 7, 2013, was re-encountered healthy in a recreational shrimp net in South Carolina on June 18, 2014, more than a year after release. (2) Turtle 13–021, originally stranding in late 2013 and released on April 22, 2014, was re-encountered healthy during a trawl for dredging mitigation in Georgia on March 9, 2018, nearly four years after release.

### Initial hormone concentrations

**Fig 1:**
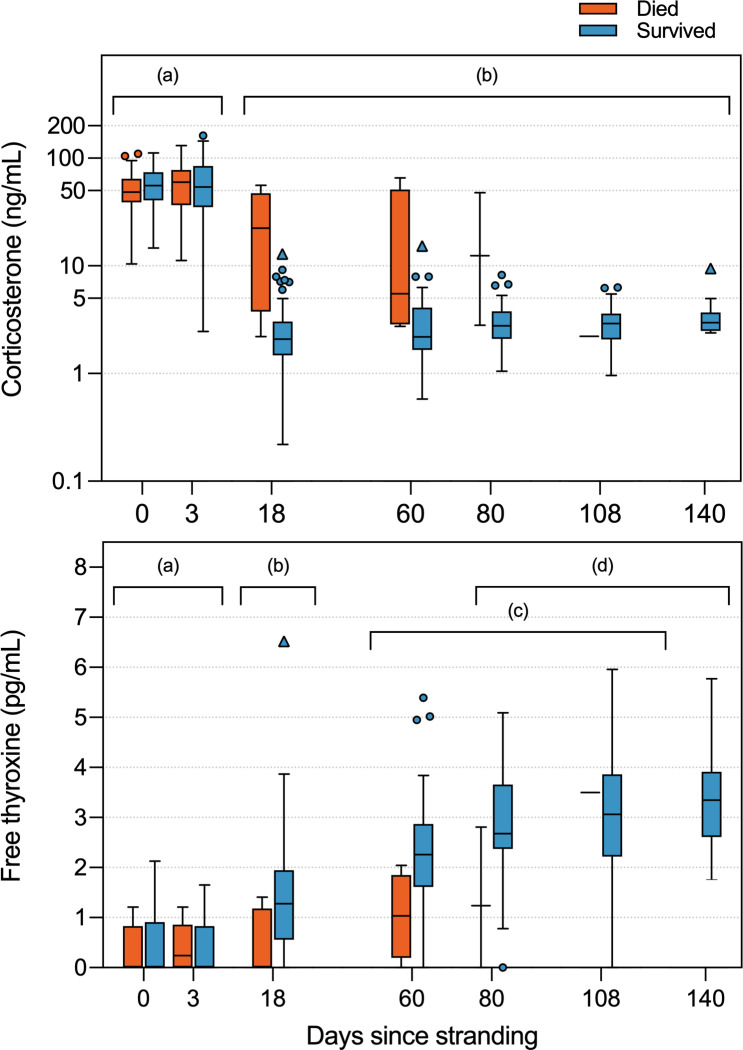
Corticosterone (ng/mL, plotted on a logarithmic scale; top panel) and free thyroxine concentrations (pg/mL; bottom panel) in plasma of Kemp’s ridley sea turtles, following stranding (day 0) and day of rehabilitation (up to day 140) from cold-stunning (n = 106 turtles, involving a total of 548 plasma samples). Turtles that survived to be released into the wild are shown in blue; turtles that did not survive are shown in orange. NB: For turtles that died, sample sizes were limited for days 80 (n = 3), 108 (n = 1), and 140 (n = 0). Boxplots represent median value, encompassed by 25th and 75th percentiles, and whiskers delineate 1.5 interquartile range. Outliers are marked with an open circle and extreme outliers with an open triangle. Different letters denote significantly different groups at p < 0.05.

In all turtles, Cort was at its peak during the first week, i.e., the day 0–1 admission sample (57.4 ± 23.2 ng/mL; *post hoc* comparisons all p < 0.001) and the day 3 sample (62.9 ± 34.6 ng/mL; all p < 0.001), with the day 3 sample typically having the highest Cort recorded for each turtle (Fig 1). On day 3, 17% of turtles had Cort >100 ng/mL, with one turtle setting a new maximal record for this species of 161.79 ng/mL (this turtle survived). Conversely, fT4 was extremely low in the first week (day 0–1 and day 3 sample, 0.4 ± 0.1 pg/mL; p < 0.001), and in fact was often undetectable (58% of day 0–1 samples had undetectably low fT4) (Fig 1).

There was a significant effect of year on initial Cort concentrations (*F*_2,98_ = 5.86, p = 0.004). The fall 2014 stranding season, which had a record-breaking number of turtle admissions (Year 3 of the study) was characterized by higher Cort levels at admission (70.4 ± 4.0 ng/mL) compared to each of the other two study years (S2 Table). Specifically, Cort concentrations in 2012 (52.6 ± 3.1 ng/mL) and in 2013 (48.7 ± 4.0 ng/mL) were each significantly lower than Cort concentrations in 2014 (post hoc comparisons: 2012 v 2014, *p* = 0.006; 2013 vs 2014, *p* < 0.001), while 2012 and 2013 did not differ significantly from each other (*p* = 0.96). However, no significant differences in fT4 concentrations were observed between study years (*F*_2,98_ = 1.29, *p* = 0.28; S2 Table).

### Patterns of hormones across rehabilitation

As expected, Cort concentration of individual turtles was significantly affected by time in rehabilitation (*F*_6,99.8_ = 565.74, p < 0.001; Fig 1, S1 Table). By day 18, at which point turtles had been swimming full-time in tanks for at least a week, Cort had dropped by an order of magnitude from the day 3 peak (Fig 1, S1 Table), with 87.95% of day 18 samples now with Cort in the presumed-normal range of < 5 ng/mL, and only 12.05% with Cort still higher than 5 ng/mL. Though there were some gradual declines from day 18 onwards, there were no further statistically significant changes in Cort with any subsequent time points (*post hoc* comparisons for days 80, 108 and 140, all *p* > 0.05), with mean Cort from day 18 or later of 3.9 ± 6.8 ng/mL (95% confidence interval range = 3.12–4.74 ng/mL) (Fig 1, S1 Table).

In contrast, fT4 showed an opposite pattern to Cort over time (*F*_6,96.3_ = 145.30, p < 0.001; Fig 1, S1 Table), significantly increasing by day 18 (p < 0.001) from its initial low, often undetectable, concentrations. Unlike Cort, fT4 continued to change after day 18, with additional significant elevations by day 60 (p < 0.001), and day 80 (p = 0.001), after which turtles appeared to maintain steady and maximal fT4 concentrations through the end of the study period at day 140 (*p* > 0.05). Free-thyroxine concentrations recorded in turtles during the later time points (*post hoc* comparisons for day 80, 108 and 140; all *p *> 0.05) averaged 3.0 ± 1.1 pg/mL (95% confidence interval range = 2.8 − 3.2 pg/mL).

### Hormones and mortality

**Fig 2:**
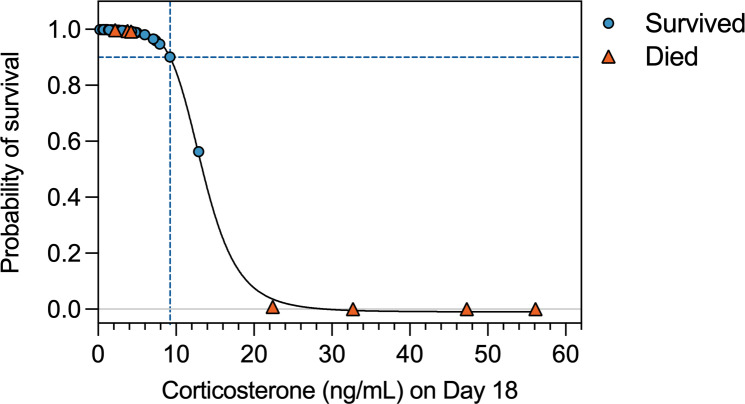
Survival curve for Kemp’s ridley turtles in relation to corticosterone concentrations (ng/mL) on day 18 after stranding. Blue circles = turtles that survived and were released to sea; orange triangles = turtles that did not survive. Dotted lines indicate maximum survival probabilities (>90%) at corticosterone concentrations below 9.2 ng/mL, with turtle survivorship exponentially diminishing with increasing corticosterone levels above 9.2 ng/mL.

Initial Cort on admission (day 0–1) and very early in rehabilitation (day 3) were not predictive of subsequent turtle mortality (Wald statistic = 0.66, p = 0.42 and Wald statistic = 0.001, p = 0.98, respectively). However, the Cort concentration on day 18 was predictive of late mortality (p = 0.02; Table 2 and Fig 2), with each additional 1 ng/ml increase in corticosterone at day 18 (e.g., from 10 ng/ml to 11 ng/ml) associated with a 21% decrease in the odds of the turtle surviving to be released into the wild. The survival probability curve indicated that turtles with low corticosterone concentrations by day 18 were in the steady phase of the curve, with maximum survival probabilities (> 90%) for individuals with Cort below 9.2 ng/mL. An inflection point occurs at 9.2 ng/mL, such that turtles with Cort >9.2 ng/mL on day 18 have a greatly increased risk of mortality (Fig 2). In contrast, fT4 was not a significant predictor of survival outcomes for cold-stunned turtles during any time point examined (p < 0.05 at 0, 3 and 18 d time points).

**Table 2:** Summary of model performance for predicting turtle survival probability based on hormone concentrations at different time points, reporting Omnibus test (*p*-value < 0.05 indicates a significant model), Akaike Information Criterion (AIC) (lower value is better), −2 Log likelihood (−2LL) (lower value is better); and Nagelkerke *R*^2^ (higher value is better).

	Model summary			
Hormone predictor variables (Cort and fT4)	Omnibus test (*p*-value)	AIC	−2LL	Nagelkerke *R*^2^
Day 0–1	χ2(2) = 0.69, *p* = 0.71	119.696	115.696	0.010
Day 3	χ2(2) = 0.04, *p* = 0.98	72.864	68.864	0.001
Day 18	χ2(2) = 22.28, *p* < 0.001	33.731	25.731	0.536

### Body mass

**Fig 3:**
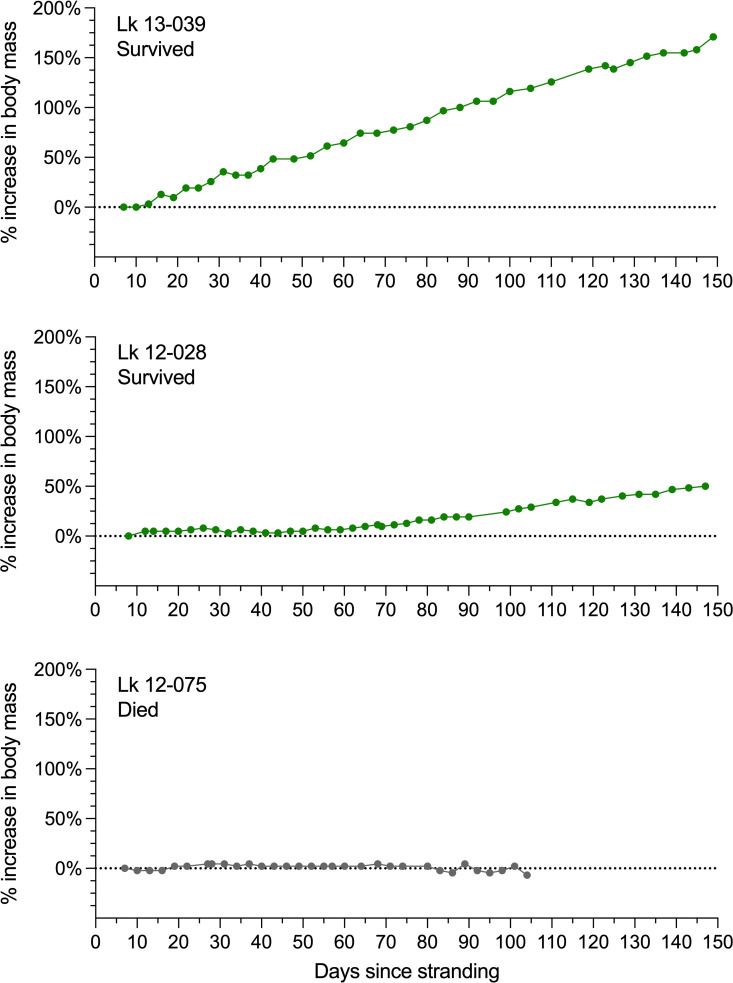
Examples of gain in body mass over time in three individual Kemp’s ridley turtles expressed as percentage increase above admission body mass (dotted line). Top, example of steady mass gain; middle, example of no gain in body mass for an initial ~2mo, followed by eventual gain at a relatively slow rate; bottom, example of no gain in body mass for >3 mo (this turtle eventually died).

Mean initial body mass did not vary by year of admission (*F*_*2,77*_ = 0.77, p = 0.47; S2 Table). Initial mass and subsequent gain in mass of turtles were negatively correlated (*r = *−0.42, p < 0.001), such that turtles with lower initial body masses typically grew faster. Turtles showed marked individual variation in rate of gain in body mass. Most turtles demonstrated stable body mass for the first 1–2 weeks, after which there was a rapid acceleration of mass gain, and then a fairly consistent rate of mass gain for the remainder of rehabilitation (i.e., straight slope; example shown in top panel of Fig 3), with mean % mass gain (from day 7 to day 60) of 21.51 ± 11.61% for surviving turtles. However, some turtles showed markedly slower growth or even no growth (examples in lower panels of Fig 3).

Of the turtles that died, few survived long enough to allow calculation of mass gain and comparison to surviving turtles. Only three survived to at least day 60 (demonstrating 0%, 2% and 7% mass gain from day 7 to day 60), of which only two survived for later mass measurements. Of these two longest-surviving turtles in the mortality group, one (Lk 12–075, shown at bottom of Fig 3) maintained approximately its initial body mass (± 2%) for the entire period of rehabilitation up to day 101, finally dying on day 104 at −6% body mass. The other case (Lk 12–085) initially showed a very slow increase in body mass for approximately three months, eventually reaching ~10% above initial body mass by day 99, at which the turtle’s mass plateaued until death at day 157.

Admission hormone concentrations of day 0–1 (i.e., when all turtles had very high Cort and very low fT4) were not related to subsequent mass gain (Cort: *r* = −0.21, *p* = 0.08 and fT4: *r *= 0.10, *p* = 0.44). However, other hormone time points showed significant relationships with mass gain, as follows: Cort at day 3 (*r* = −0.32, *p* = 0.01), day 18 (*r* = −0.38, *p* = 0.001) and day 60 (*r* = −0.32, *p* = 0.007) was negatively correlated with mass gain, while fT4 at day 18 (*r* = 0.34, *p* = 0.004) and day 60 (*r* = 0.24, *p* = 0.048) was positively correlated to mass gain.

**Fig 4:**
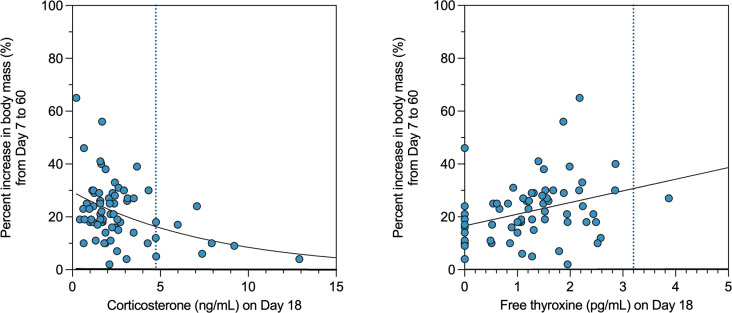
Mass gain (percent increase in body mass from day 7 to day 60; y-axis) compared to plasma hormone concentrations at day 18 (x-axis) for corticosterone (left) or thyroxine (right) in juvenile Kemp’s ridley sea turtles. Each point represents one turtle. Solid line indicates the best fit curve estimation for observed corticosterone (logistic curve line, p < 0.001) and thyroxine concentrations (linear line, p = 0.004). Vertical dotted line indicates the upper value of 95% confidence interval range as determined in this study (corticosterone, 4.74 ng/mL; and thyroxine, 3.2 pg/mL).

In examination of the day 18 time point specifically as a potential predictor of later outcomes, individual mass gain in turtles by day 60 was best explained by an endocrine profile on day 18 of low Cort (Wald statistic = 13.99, df = 1, *p* < 0.001) and high fT4 (Wald statistic = 5.36, df = 1, *p* = 0.02). Curve estimation showed that the relationship between mass gain and day 18 Cort was best explained by a non-linear logistic model (*R*^2^ = 0.21, *F*_1,67_ = 17.75, *p* < 0.001; Fig 4, left panel), whereas a simple linear relationship fitted the observed data for mass gain and day-18 thyroxine (*R*^2^ = 0.12, *F*_1,67_ = 8.80, *p* = 0.004; Fig 4, right panel). In contrast, consistent feeding (Wald statistic = 0.43, df = 1, *p* = 0.51), mean daily g of ingested food (Wald statistic = 2.66, df = 1, *p* = 0.10) and consistent activity (Wald statistic = 0.32, df = 1, *p* = 0.58), all assessed across the week prior to day 18 (i.e., feeding and activity of days 11–17), were not significant predictors of subsequent mass gain.

### Relationship of hormones with feeding and activity

Cort concentrations on day 18 were significantly and negatively associated with the amount of food ingested per day during the week prior (*F*_1,76_ = 4.52, p = 0.04). For example, turtles that did not eat during the seven days prior to blood measurement had elevated Cort (18.7 ± 20.1 ng/mL, range = 2.6 − 56.1 ng/mL) compared to concentrations of Cort of turtles that ingested any food (Cort 2.5 ± 2.0 ng/mL, range = 0.22 − 12.9 ng/mL; ingested food, 29.9 ± 17.4 g per day, range = 0.6 − 89.7 g). However, Cort concentrations showed no apparent relationship with consistency of feeding behavior (*F*_1,76_ = 1.16, p = 0.29) or activity levels (*F*_1,76_ = 1.01, p = 0.32). By contrast, fT4 concentrations of day 18 were significantly and positively associated with consistent feeding behavior during the prior week (*F*_1,76_ = 4.51, p = 0.04; fT4 of turtles that ate every day = 1.8 ± 0.2 pg/mL; fT4 of turtles that did not eat every day = 0.8 ± 0.1 pg/mL). However, fT4 was not related to the amount of food ingested per day (*F*_1,76_ = 1.33, p = 0.25) or activity (*F*_1,76_ = 2.43, p = 0.12).

## Discussion

Results from our three-year prospective study confirm and extend previous findings that corticosterone is extremely high and thyroxine very low in cold-stunned juvenile Kemp’s ridley sea turtles upon admission, with the two hormones inversely correlated. Cort then drops to presumed-normal concentrations after approximately two weeks into rehabilitation in most turtles, but thyroxine continues to elevate for up to 80 days of rehabilitation, a finding not apparent from earlier studies. Endocrine status showed consistent and significant relationships with mortality, mass gain, and feeding, with the general pattern that turtles with low Cort and high fT4 tend to eat well, gain weight steadily, and are more likely to survive. Notably, endocrine status at day 18 can predict later mortality and growth trends up to day 60 of rehabilitation, indicating that endocrine measures might be useful prognostic indicators for cold-stunned turtles over at least a two-month time span.

### Changes in hormones over time

Corticosterone’s pattern of very high concentrations upon admission, followed by a dramatic drop by two weeks, has been reported previously [10]. Unexpectedly, however, this study revealed that Cort increases further from day 0 to day 3, with the day 3 concentration representing the highest Cort yet reported for these species. On day 3, 17% of turtles in our study had Cort concentrations more than twenty times higher than presumed-normal (>100 ng/mL, as compared to presumed-normal of <5 ng/mL), with a maximum of ~32 times higher than presumed-normal (161.79 ng/mL; this turtle survived). Remarkably, these extraordinarily high Cort concentrations during the first week of rehabilitation do not appear to affect the turtles’ prognoses. Stress physiology literature from other taxa (e.g., birds) shows consistently that brief spikes in Cort are not only survivable but are likely adaptive, while it is prolonged elevations (even if much more moderately elevated) that are more likely to be associated with clinical concerns and long-term negative impacts (reviewed in [24]). The question remains, however, why Cort increases at day 3 as compared to day 0–1. One possibility is that turtles may become more aware that they are now in a captive environment. Cold-stunned Kemp’s ridleys typically arrive at the NEAq so chilled as to often be completely immobile and unreactive, to such a degree that in some cases it can be difficult to determine if they are alive, and they typically become much more alert as they are warmed during days 0–3. Thus, turtles may “wake up” to an unfamiliar clinical environment, where additional stressors — such as required medical procedures and associated handling — have been added to the stressors of their previous stranding experience and underlying pathologic conditions. Additional possibilities for the additional elevation in day 3 include possible effects on adrenal activity due to increased body temperature, or overcompensation of gluconeogenic mechanisms (e.g., an “overshoot” of adrenal activity in the process of mobilizing energy for survival; see [9]). Encouragingly, by just two weeks later, turtles seem to have largely adjusted to the clinical environment; in 88% of turtles, Cort reduced dramatically by day 18, to <5 ng/mL, and this study shows that Cort does not alter significantly thereafter. This finding supports earlier estimates that the < 5 ng/mL range can be presumed “normal,” or at least near-normal, for this species (see also [20]), and indeed our study shows that turtles with Cort <5 ng/mL are not at increased risk of mortality. However, growth rate data, discussed further below, indicate that even within this “presumed-normal” range of <5 ng/mL, there may be meaningful physiological differences between turtles on the lower end of that range vs. the higher end.

As expected, fT4 showed opposite patterns, being undetectable or nearly undetectable upon admission and rising to detectable levels of approximately ~1 pg/mL by two weeks. Unlike Cort, fT4 then continued to rise for at least two more months, finally plateauing at approximately the third month of rehabilitation at ~3 pg/mL. This is three times higher than our prior estimate of “presumed-normal” fT4 (originally derived from day 18 samples; [10]). It is possible that the turtle thyroid axis does not fully return to physiologically normal activity until 2–3 months of rehabilitation. Another possibility is that the experience of cold-stunning — i.e., of being chilled below functional temperatures — may induce a subsequent increase in thyroid activity, perhaps an adaptive response aimed at avoiding future episodes of cold-stunning. Alternatively, this may be a normal seasonal pattern; the day 80 time point at which fT4 stabilizes typically occurs at a specific time of year, usually January or February, and it may be that fT4 normally elevates in winter in juveniles of this species. However, studies of adult Kemp’s ridleys show a drop in T4 in January in females, and no change in males [53], and other sea turtles show no consistent evidence of seasonality in T4 (e.g., no seasonal pattern in the green turtle, *Chelonia mydas*; [54]). While these details of why and how fT4 continues to slowly rise over several months are perhaps not of clinical relevance, future studies of sea turtle thyroid hormone, body temperature, activity and metabolic rate could be informative for understanding the role of the thyroid gland in ectotherms, and exploring the degree to which ectotherms are able to adjust their metabolic rate across seasons or in response to environmental challenges (see also [55,56]). Given the observed positive correlations of fT4 with mass gain and feeding behavior, clinicians at NEAq sometimes prescribe thyroxine supplementation for chronically anorexic turtles, and future studies will assess the efficacy of this practice.

### Corticosterone and mortality

In this study, Cort was found to be a significant predictor of late mortality of cold-stunned turtles, specifically mortality that occurs after two weeks of rehabilitation. Late mortality is relatively rare in the NEAq clinic (most mortality of cold-stunned turtles occurs in the first week), but some losses do occur late in rehabilitation due to pathologic conditions such as sepsis and pneumonia, and it may be valuable to identify prognostic indicators of such cases. Corticosterone has been documented to predict mortality in multiple other vertebrates, including ectotherms such as Galápagos marine iguanas (*Amblyrhynchus cristatus*; [57]) and eastern fence lizards (*Sceloporus undulatus*; [58]) as well as various endotherms (e.g., [59–61]). However, the strength and even the direction of this relationship varies with context, such as mild vs. extreme elevations in corticosterone, prior individual history, current health status, and short- vs. long-term survival (see examples in [62–64]). Our study suggests that in the context of veterinary management of cold-stunned sea turtles, a single routine measurement of plasma corticosterone on or near day 18 — a time point at which a blood sample is often taken anyway — could identify turtles-at-risk. Some such turtles-at-risk may have been already identified as such by clinicians based on physical and behavioral observations, clinical pathologic data, and radiography, but some may not; further, stress assessment literature suggests that multiple and redundant metrics of stress and health are desirable (e.g., [24]), i.e., endocrine data can be regarded as complementary to other clinical data. Additionally, in some clinical contexts (i.e., particularly large numbers of strandings), individual monitoring is not always possible, and detailed clinical status may not be available for all turtles. In such a context, endocrine information could then be a valuable source of supplemental information.

### Corticosterone and growth

Juvenile sea turtles normally grow steadily and rapidly, and it is believed that this high growth rate is important to help juveniles grow “beyond bite-size”, i.e., high growth rate helps young turtles attain a large enough body size that their carapace can increasingly provide an effective barrier to gape-limited aquatic predators. Growth rate is not often assessed in sea turtle rehabilitation, with most published studies of clinical outcomes focusing solely on survival and release back to sea. However, a released turtle that is unusually small for its age may in theory be at heightened risk of predation (i.e., compared to same-age cohorts that had normal growth rate). Our study shows that in a rehabilitation context, endocrine status at day 18 is associated with the turtle’s total growth (increase in body mass) over at least two months of rehabilitation (day 7 to day 60). Further, though sample size for late mortalities was very small, the few late-mortality cases that could be evaluated for long-term patterns in growth demonstrated remarkably slow or no growth in body mass, despite their juvenile age class, a life stage normally characterized by steady growth. To some degree this relationship may merely reflect poor clinical status (i.e., poor clinical status may be driving both the high Cort and the unusually low growth rate). However, examination of Fig 4 (left panel) reveals that even if outliers with unusually high Cort are excluded, the remaining dataset, which largely consists of turtles thought to be in good clinical status, still illustrates a fairly consistent relationship in which even relatively subtle elevations in Cort are associated with lower rates of gain in body mass. This finding is perhaps unsurprising given the well-documented phenomenon of glucocorticoids inhibiting growth in vertebrates generally (e.g., [65,66]), and it suggests that even within the “presumed-normal” Cort range, lower Cort (e.g., 2 ng/mL rather than 5 ng/mL) may be better. Put in other words, normal may not be the same thing as optimal. Thus, continued efforts to minimize stress in the clinical context (where possible) may be beneficial, even for those turtles that are clinically stable and in good condition.

Our study cannot identify the mechanism by which Cort may affect growth, or even the direction of causality. However, controlled experiments in other taxa show that stress-induced elevations in Cort directly inhibit release of both growth hormone from the anterior pituitary and insulin-like growth factor 1 from the liver, important mediators of somatic growth in vertebrates (reviewed in [67–69]). Alternatively, Cort might reduce growth in turtles by inducing behavioral changes that result in a shift in energetic balance (e.g., turtle moving more and/or eating less), or possibly shifting basal metabolic rate; yet, we found no relationship of Cort with activity, and a positive association between Cort and amount of food eaten per day, and, further, no relationship of growth with fT4 (a major determinant of basal metabolic rate; [38–41,55,56,70]). It should also be noted that our primary metric of growth, gain in body mass, cannot distinguish between lean mass, growth in body dimensions (carapace length and width), and changes in fat depots. Anecdotally, however, none of the turtles in this study were noted by clinicians to have developed signs of obesity (e.g., bulging fat depots around the neck, shoulder girdle and pelvic girdle), and at release all turtles were assessed in final pre-release veterinary examinations as in good health and in normal body condition. Future study of carapace length and width in sea turtles in rehabilitation may be useful for determining expected growth rates in body dimensions. We recommend further investigation of growth rate in rehabilitated sea turtles, including development of species-specific screening metrics for expected growth at certain time points. More detailed studies of energetic balance would be informative, for example the use of accelerometer data to inform a more complete understanding of turtle activity and associated caloric expenditure. It would likely also be informative to continue and expand efforts at telemetric monitoring of rehabilitated turtles after release, to investigate whether turtles that grow more slowly during rehabilitation might be at increased risk of mortality after release.

### Year effects

One year of our study, Year 3 (fall 2014 stranding season) was marked by an extraordinary increase in the number of stranded sea turtles discovered on Cape Cod, with nearly an order of magnitude increase both in stranding numbers (many of which did not survive to admission) and in clinical caseload. Though we found no significant increase in mortality in turtles admitted to the NEAq clinic in Year 3, Cort at admission was significantly higher in Year 3. This high initial Cort could possibly be related to whatever prior weather, wind, or oceanographic conditions resulted in the marked increase in strandings (i.e., turtles may have been stressed or in poor condition for some days or weeks in Cape Cod Bay before the strandings occurred). It is believed that there were not unusual delays in transporting turtles from the stranding location to the NEAq clinic that could explain the higher Cort on admission in Year 3, but it is possible that high caseload and associated alterations in clinical activities (e.g., more people, vehicles, noise, etc.) may have influenced turtle Cort concentrations. fT4, however, was not different in this year compared to other years – likely because fT4 is already minimal at this time point even in a typical year (and often is below the detectability limit of the assay). In sum, years with unusually high strandings may present clinical challenges that involve not just a high number of turtles, but turtles being, on average, more stressed and in poorer condition as compared to other years.

### Study limitations

Our study design was limited by multiple factors, including caseload, clinical logistics, and important animal-welfare and endangered-species management concerns that required that our clinical focus remain squarely on maximizing each individual’s chances of recovery and release to sea. Thus, for example, blood sampling timing had to accommodate existing clinical schedules, and certain controlled experiments were not possible (i.e., we could not experimentally induce cold-stunning or experimentally administer exogenous Cort). Further, sample sizes for late mortality were small; this is of course desirable from a clinical perspective, but results in low statistical power for certain of our findings. The inherently attritional nature of the dataset results in progressive reductions in statistical power for each time point (e.g., healthy turtles began to leave the study after day 60 due to release back to sea, while turtles in poor condition disappear from later time points due to mortality). We encourage future research to fill some of these gaps and improve understanding. For example, a multi-year prospective study targeting later time points, as well as routine collection of certain additional data (body mass, carapace dimension, etc.) across multiple years would help increase sample sizes and improve statistical power.

## Conclusions

This three-year study revealed that cold-stunned sea turtles undergo long-term changes in endocrine status that can continue for several months after initial stranding. While small sample sizes for certain groups (e.g., relatively few cases of late mortality) warrant caution in interpretation, the significant correlations of Cort with mortality, and of both hormones with long-term mass gain, suggest potential relevance of regular endocrine monitoring for clinical care. Many data gaps still need filling, however, such as expected rates of gain in body mass, prognosis for those turtles with unusually slow growth, potential effects of subtle elevations in Cort, and evaluation of thyroxine supplementation as a potential therapeutic intervention for anorexic turtles. Our study demonstrates that even when turtles survive stranding, continued stress may have long-term impacts on growth and body size; thus, it would be useful to determine whether poor mass gain in captivity entails any significant risks for turtles after release, e.g., greater risk of depredation of turtles released at a body mass that may be “small for their age”. The endocrine assays described here could provide screening tools that might assist clinicians and researchers in identifying turtles at risk of poor growth or of mortality, particularly in those clinical or research situations in which regular clinical evaluation may not be possible. As sea surface temperatures continue to change and mass sea turtle strandings continue to occur, continued study of glucocorticoids, thyroid hormones, and other physiological biomarkers of recovery in sea turtles may be useful for clinical care, research and management of sea turtles generally.

## Supporting information

S1 Table Hormone means across sampling time points. Summary of corticosterone (ng/mL) and thyroxine concentration (pg/mL) in Kemp’s ridley turtles, following days since stranding and rehabilitation in hospital (total *n* = 106 turtles).
(DOCX)

S2 Table First-week data divided by study year. Descriptive statistics for initial body mass (kg), initial corticosterone concentrations (ng/mL) and initial thyroxine concentrations (pg/mL) in Kemp’s ridley turtles in their first week after admission, in each of the three study years (total *n* = 106 turtles). Year 3 (2014) had a record-breaking number of stranded turtles.
(DOCX)

S3 Table Full dataset. Complete dataset of all data analyzed in this study, including turtle identification numbers, stranding date, corticosterone, thyroxine, gain in body mass, feeding, and activity.
(XLSX)
