# Supplementary material for: Relationships of corticosterone and thyroxine with mortality, mass gain, feeding and activity in Kemp’s ridley sea turtles (Lepidochelys kempii) recovering from cold-stunning
Source: PLoS One. 2025 Jun 18;20(6):e0325265. doi: 10.1371/journal.pone.0325265 (PMC12176108; doi:10.1371/journal.pone.0325265)
Supplement: S1 Table — Summary of corticosterone (ng/mL) and thyroxine concentration (pg/mL) in Kemp’s ridley turtles, following days since stranding and rehabilitation in hospital (total n = 106 turtles). (DOCX) [file pone.0325265.s001.docx]

**S1 Table. Hormone means across sampling time points.** Summary of corticosterone (ng/mL) and thyroxine concentration (pg/mL) in Kemp’s ridley turtles, following days since stranding and rehabilitation in hospital (total n = 106 turtle
