# Supplementary material for: Relationships of corticosterone and thyroxine with mortality, mass gain, feeding and activity in Kemp’s ridley sea turtles (Lepidochelys kempii) recovering from cold-stunning
Source: PLoS One. 2025 Jun 18;20(6):e0325265. doi: 10.1371/journal.pone.0325265 (PMC12176108; doi:10.1371/journal.pone.0325265)
Supplement: S2 Table — Descriptive statistics for initial body mass (kg), initial corticosterone concentrations (ng/mL) and initial thyroxine concentrations (pg/mL) in Kemp’s ridley turtles in their first week after admission, in each of the three study years (total n = 106 turtles). Year 3 (2014) had a record-breaking number of stranded turtles. (DOCX) [file pone.0325265.s002.docx]

**S2 Table. First-week data divided by study year.** Descriptive statistics for initial body mass (kg), initial corticosterone concentrations (ng/mL) and initial thyroxine concentrations (pg/mL) in Kemp’s ridley turtles in their first week after admission, in each of the three study years (total n = up to 106 turtles). Year 3 (2014-15) had a record-breaking number of stranded turtles.
